# A Novel Method for Identifying Essential Proteins Based on Non-negative Matrix Tri-Factorization

**DOI:** 10.3389/fgene.2021.709660

**Published:** 2021-08-06

**Authors:** Zhihong Zhang, Meiping Jiang, Dongjie Wu, Wang Zhang, Wei Yan, Xilong Qu

**Affiliations:** ^1^College of Computer Engineering and Applied Mathematics, Changsha University, Changsha, China; ^2^Department of Ultrasound, Hunan Provincial Maternal and Child Health Care Hospital, Changsha, China; ^3^School of Information Technology and Management, Hunan University of Finance and Economics, Changsha, China; ^4^Department of Banking and Finance, Monash University, Clayton, VIC, Australia; ^5^Department of Optoelectronic Engineering, Jinan University, Guangzhou, China; ^6^Hunan Provincial Key Laboratory of Finance and Economics Big Data Science and Technology, Hunan University of Finance and Economics, Changsha, China

**Keywords:** non-negative matrix factorization, protein-protein interaction, essential protein, PageRank, network

## Abstract

Identification of essential proteins is very important for understanding the basic requirements to sustain a living organism. In recent years, there has been an increasing interest in using computational methods to predict essential proteins based on protein–protein interaction (PPI) networks or fusing multiple biological information. However, it has been observed that existing PPI data have false-negative and false-positive data. The fusion of multiple biological information can reduce the influence of false data in PPI, but inevitably more noise data will be produced at the same time. In this article, we proposed a novel non-negative matrix tri-factorization (NMTF)-based model (NTMEP) to predict essential proteins. Firstly, a weighted PPI network is established only using the topology features of the network, so as to avoid more noise. To reduce the influence of false data (existing in PPI network) on performance of identify essential proteins, the NMTF technique, as a widely used recommendation algorithm, is performed to reconstruct a most optimized PPI network with more potential protein–protein interactions. Then, we use the PageRank algorithm to compute the final ranking score of each protein, in which subcellular localization and homologous information of proteins were used to calculate the initial scores. In addition, extensive experiments are performed on the publicly available datasets and the results indicate that our NTMEP model has better performance in predicting essential proteins against the start-of-the-art method. In this investigation, we demonstrated that the introduction of non-negative matrix tri-factorization technology can effectively improve the condition of the protein–protein interaction network, so as to reduce the negative impact of noise on the prediction. At the same time, this finding provides a more novel angle of view for other applications based on protein–protein interaction networks.

## Introduction

Essential proteins play an indispensable role in the survival of organisms, and the criticality of proteins is mainly determined by their biological functions. Studies have shown that essential proteins have abundant functions such as translation, transcription, and replication (Glass et al., 2009). The prediction of essential proteins can apply the important reference information of biology and medicine, which has a wide application prospect in the fields of disease diagnosis and drug design. Currently, researchers have proposed a variety of biological methods to identify essential proteins, such as single-gene knockout (Kobayashi et al., 2003). However, these experimental methods have some limitations such as high cost and long time consumption. Therefore, it is urgent to improve the prediction performance of the computational method to identify essential proteins.

In recent years, researchers have proposed many computational methods to identify essential proteins relying on different ideas and technologies. Researchers have proposed many classic algorithms for predicting essential proteins based on PPI network topological characteristics, such as degree centrality (DC) (Hahn and Kern, 2005), information centrality (IC) (Björnsdottir, 2001), closeness centrality (CC) (Wuchty and Stadler, 2003), betweenness centrality (BC) (Joy et al., 2014), subgraph centrality (SC) (Estrada and Rodríguez-Velázquez, 2005), and sum of edge clustering coefficient centrality (NC) (Wang et al., 2012). Li et al. (2018) found that in the PPI network, the frequency of essential proteins in triangular structures is significantly higher than that of non-essential proteins. Based on this research discovery, they proposed a new measure of pure Centrality-Neighborhood Closeness Centrality (NCC). Although this type of approach allows direct identification of essential proteins in the absence of known essential proteins, there are limitations to these approaches. First, the existing PPI data are incomplete with a large number of false positives and false negatives, affecting the accuracy of predicting essential proteins. Second, most of these methods just use the topological properties of the network while ignoring other properties of essential proteins.

In order to make up for the limitations of incomplete protein interaction networks, many research groups have combined PPI networks with other biological information in recent years to improve the accuracy of essential protein identification. Tew et al. (2007) proposed a novel method called NFC, which defines the functional similarity between two proteins based on the GO term similarity and scores the protein based on the sum of the functional similarity between the protein and its neighboring proteins. Zhang et al. (2018) proposed an essential protein prediction method named TEO by combining the network topology characteristics, gene expression information, and GO annotation information. A weighted protein interaction network was established by calculating the Edge Clustering Coefficient (ECC), Pearson Correlation Coefficient (PCC), and functional similarity, so as to realize essential protein recognition. Lei et al. (2019) proposed an essential protein identification method called RWEP. Firstly, a weighted PPI network was established using network topology, gene expression, and GO annotations; then, each protein in the network was identified according to subcellular localization and protein complexes. Finally, the restart random walk algorithm is used to iteratively calculate the protein score in the weighted network. Due to the strong clustering of essential proteins, Ren et al. (2011) proposed a new centrality method that combines PPI network topology and protein complex information to identify essential proteins. By fusing the topological feature of PPI networks and gene expression information, Zhang et al. (2013) and Li et al. (2012) proposed two different models to predict essential proteins, called CoEWC and PeC, respectively. Based on the modular characteristics of essential proteins, Zhao et al. (2014) proposed an essential protein identification method called POEM. Based on the network topological characteristics and gene expression information, a highly reliable weighted network was established, and on this basis, overlapping functional modules with high cohesion and low coupling were dug. Finally, scores were calculated according to the weighted density of the modules to which the proteins belong, so as to realize the identification of essential proteins. Peng et al. (2012) considered that essential proteins were more conservative than non-essential proteins and often combined with each other. They proposed an iterative method ION that combines direct homology and PPI networks to predict essential proteins. The probability transfer matrix was established by using the edge clustering coefficient (ECC) and interaction network, and the initial score vector of protein was established by using homology information. According to the similarities of active PPI networks of each time, Peng et al. (Zhang et al., 2019) established a novel PPI network. Then, based on this network and orthologous information of protein, they developed a dynamic protein–protein interaction network-based model called FDP. Zhong et al. (2021) proposed a new measure method called JDC, which offers a dynamic threshold method to binarize gene expression data and combines Jaccard similarity index and degree centrality to predict essential proteins. However, the methods based on multisource data are relatively simple. It not only will conceal the complex relationship between the multisource data but also may introduce artificial noise.

In this article, we utilize non-negative matrix tri-factorization (NMTF) to deal with the challenges introduced above and propose a novel method named NTMEP for identifying essential proteins. NTMEP focuses on the following three important aspects. First, it is well known that the multiple kinds of biological data about proteins can be integrated to construct a weighted PPI network with similar functions. As a result, the more different types of data are used, the more artificial noise is produced inevitably. Considering this problem, NTMEP constructs the weighted PPI by using original protein–protein interaction information merely. Second, the NMTF algorithm is extensively used for many applications in pattern recognition, text mining, DNA gene expressions, and so on. This is also extended to community detection and the recommendation system. Hence, to mine more potential protein–protein associations, the NMTF algorithm is introduced in our progress. It takes the internal possibility of associations between proteins into account, which contributes to generation of a more reliable prediction model that excludes the noisy candidates. Third, distinct from previous approaches, we employ homologous and subcellular localization information in the course of ranking proteins, which can improve the accuracy of predicting essential proteins effectively.

## Materials and Methods

Our purpose is to develop a novel method which can improve the accuracy of predicting essential proteins. We firstly constructed a weighted PPI network to represent the complex relationships between proteins. Moreover, a novel prediction method based on NMTF was proposed specifically for the network to find the potential associations between proteins. Finally, the PageRank algorithm was performed to identify the essential protein candidates by integrating subcellular localization and homologous information.

Let *G*(*V*, *E*) be the PPI network that contains node set *V* = (*p*_1_, *p*_2_, …, *p*_*n*_) (*n* is the number of proteins) and edge set *E* = [(*p*_1_, *p*_2_, *w*_1_), (*p*_2_, *p*_3_, *w*_2_), …, (*p*_*i*_, *p*_*j*_, *w_*m*_*)] where (*p*_*i*_, *p*_*j*_, *w_*m*_*) is the interaction between protein *p*_*i*_ and *p*_*j*_ with weighted value *w*_*m*_ which was set to 1 in original protein–protein interaction information.

### Protein Association Measurement

In this subsection, a weighted PPI network was constructed in which the association value of two proteins would be calculated based on their topological characteristics. In analyzing the topological characteristics of PPI networks, researchers have found that the PPI networks are one kind of small-world and scale-free network. Therefore, the topological features of the PPI network can be used to predict essential proteins. In recent years, the item of common neighbors of two proteins in the PPI network has been used in many prediction algorithms to realize the task of predicting essential proteins. They demonstrate that the more common neighbors exist between two proteins, the more deeply is the association they have with other. In this article, if proteins *p*_*i*_ and *p*_*j*_ share at least one common neighbor, we assume that *p*_*i*_ and *p*_*j*_ are interacting. This kind of connection between proteins is called the co-neighbor (CoN) relationships and calculated as follows:

(1) $$\begin{array}{c} P_{CoN}\left(i,j\right)=\\ \left\{ \frac{{\left|S_{Nei}\left(i\right)⋂S_{Nei}\left(j\right)\right|}^{2}}{\left(\left|S_{Nei}\left(i\right)\right|-1\right)*\left(\left|S_{Nei}\left(j\right)\right|-1\right)}\begin{array}{c} if\left|S_{Nei}\left(i\right)\right|>1and\\ \left|S_{Nei}\left(j\right)\right|>1 \end{array}\right.\\ \;\;0\;otherwise \end{array}$$

where **S*_*Nei*_(*i*)* and **S*_*Nei*_(*j*)* present the neighborhood sets of *p*_*i*_ and *p*_*j*_, respectively. As can be seen from the above equation, the value of the CoN relationships of the two-protein range is between 0 and 1.

### Reconstruction of the Weighted PPI Network Based on NMTF

Non-negative matrix tri-factorization as a general technology takes or compresses a data matrix into a compact latent space. It has been used to model topics in text data (Hua et al., 2011), to predict cancer driver genes from clinical data (Xi et al., 2018), and to detect disease–disease associations (Žitnik et al., 2013). It is an efficient data representation technique, which has been widely used in recommender systems (Hernando et al., 2016; Luo et al., 2016). This new understanding should help to improve prediction accuracy of the essential proteins.

To take full advantage of NMTF, we perform it on the weighted PPI network (*P*_*CoN*_) to mine the potential interactions of proteins. In contrast to classic non-negative matrix factorization (Lee and Seung, 1999) where the input matrix is separated into two parts, NMTF resolves the input matrix into three latent matrices. Here, we consider that the input adjacency matrix *P*_*CoN*_ ∈ *R*^n*n^ has missing records, that is to say, the interactions between proteins have not been discovered. By using NMTF, a new matrix *Y* ∈ *R*^n*n^ containing some new records would be constructed, as follows:

(2) $$P_{CoN}\approx Y=FSG^{T}$$

Here, NMTF is designed to describe the matrix *P*_*CoN*_ ∈ *R*^n*n^ with a product of three non-negative potential matrices *F* ∈ *R*^n*k^, *S* ∈ *R*^k*k^, and *G* ∈ *R*^n*k^, while parameter *k* denotes factorization ranks and represents the number of potential vectors which form the column and row column space. For a given non-negative data matrix **P*_*CoN*_*, the issue can be solved as the following optimization problem:

(3) $$D=\min J\left(F,S,G\right)={||P_{CoN}-FSG^{T}||}_{F}^{2}$$

where ||⋅||*_*F*_* is the Frobenius norm. Since the objective function in Eq. (3) is a joint non-convex problem, we employ the rule of multiplicative iteration to solve the objective function on the basis of using auxiliary functions. The squared Frobenius norm can be written as | | *X*| | ^2^ = *Tr*(*X^*T*^X*); therefore, Eq. (3) equals to:

(4) $$D=Tr\left(P_{CoN}^{T}P_{CoN}-2P_{CoN}^{T}FSG^{T}+GS^{T}F^{T}FSG^{T}\right)$$

Its partial derivative equations for factor *F*, *S*, and *G* are as follows, respectively:

(5) $$\begin{array}{c} \frac{\partial D}{\partial F}=2FSG^{T}GS^{T}-2P_{CoN}GS^{T}\\ \frac{\partial D}{\partial S}=2F^{T}FSG^{T}G-2F^{T}P_{CoN}G\\ \frac{\partial D}{\partial G}=2GS^{T}F^{T}FS-2P_{CoN}^{T}FS \end{array}$$

It is well known that the static point can be detected using the Karush–Kuhn–Tucker (KKT) complementarity conditions. The KKT condition for factor *F* is as follows:

(6) $$\frac{\partial D}{\partial F_{ik}}F_{ik}=0$$

In this connection, the conditions are assumed to be functional if the derivative is zero:

(7) $$\begin{array}{c} {\left(FSG^{T}GS^{T}-P_{CoN}GS^{T}\right)}_{iu}F_{iu}=0\\ F_{iu}=F_{iu}\frac{{\left(P_{CoN}GS^{T}\right)}_{iu}}{{\left(FSG^{T}GS^{T}\right)}_{iu}} \end{array}$$

Similarly, the updating rules for *G* and *S* can be derived as follows:

(8) $$\begin{array}{c} G_{iu}=G_{iu}\frac{{\left(P_{CoN}^{T}FS\right)}_{iu}}{{\left(GS^{T}FFS^{T}\right)}_{iu}}\\ S_{iu}=S_{iu}\frac{{\left(F^{T}P_{CoN}G\right)}_{iu}}{{\left(F^{T}FSG^{T}G\right)}_{iu}} \end{array}$$

The multiplication iteration rules are shown as follows:

(9) $$\begin{array}{c} F_{iu}\leftarrow F_{iu}\frac{{\left(P_{CoN}GS^{T}\right)}_{iu}}{{\left(FSG^{T}GS^{T}\right)}_{iu}}\\ G_{iu}\leftarrow G_{iu}\frac{{\left(P_{CoN}^{T}FS\right)}_{iu}}{{\left(GS^{T}FFS^{T}\right)}_{iu}}\\ S_{iu}\leftarrow S_{iu}\frac{{\left(F^{T}P_{CoN}G\right)}_{iu}}{{\left(F^{T}FSG^{T}G\right)}_{iu}} \end{array}$$

From the above Eq. (9), the optimal matrix *Y*, which is closest to **P*_*CoN*_*, can be computed. Finally, to recover the symmetry of the protein–protein interactions, we transformed the matrix *Y* to a symmetrical transition probability matrix $P_{CoN}^{*}$, as follows:

(10) $$\begin{array}{c} P_{CoN}^{*}\left(i,j\right)=\{\frac{\text{max}\left(Y_{ij},Y_{ji}\right)}{\sum_{k=0}^{N}Y_{ik}},\;\sum_{k=0}^{N}Y_{ik}\neq 0\\ \;0,\;\text{else} \end{array}$$

### The NMTF-Based Model for Identifying Essential Proteins

Through the description of the above algorithm, based on the information of the original PPI network, an optimized weighted PPI network can be established. Therefore, we can use an iterative method to rank protein scores. This method mainly includes two parts: the calculation of the initial score and the calculation of the ranking score, as detailed below.

### Computation of Initial Scores

In this part, we will initially score each protein in the PPI network using homologous and subcellular localization information. Taking the *Saccharomyces cerevisiae* PPI network as an example, Tang et al. (2018) analyzed whether all the proteins in this network had direct homologous proteins in 99 reference species. They concluded that the more homologous a protein has in the reference species, the more likely it is to become a required protein. In order to obtain the given protein *p*_*i*_ in the PPI network *G* = (*V*, *E*), we mainly use the homology information to calculate the homology score (*S*_*H*_) of the protein. Among them, *S*_*H*_ (*p*_*i*_) refers to the conservative score of *p*_*i*_, and the calculation formula is as follows:

(11) $$S_{H}\left(p_{i}\right)=\frac{H\left(p_{i}\right)}{\max_{1\leq j\leq \left|V\right|}\left(H\left(p_{j}\right)\right)}$$

Among them, *H*(*p*_*i*_) refers to the number of times that the protein *p*_*i*_ has direct homologous proteins in the reference species.

We know that an important feature of proteins is subcellular localization. By studying the characteristics of protein subcellular localization, researchers (Li et al., 2016; Zhao et al., 2016; Lei et al., 2018) found that essential proteins are more likely to appear in specific subcellular locations. Based on this, we calculated the subcellular localization score (*S*_*L*_) of the protein based on the subcellular localization information. If the protein *p*_*i*_ exists in the final subcellular localization dataset *R*, then the frequency of each subcellular location *r* can be calculated by the following formula:

(12) $$OF\left(r\right)=\frac{\left|SN\left(r\right)\right|}{\max_{1\leq k\leq n}\left(\left|SN\left(k\right)\right|\right)}$$

where *SN* represents the relationship between the protein and the subcellular location data set, *SN*(*r*) refers to the number of proteins corresponding to the subcellular location *r*, and *n* is the number of subcellular locations.

Based on a fixed protein *p*_*i*_, the subcellular localization score *S*_*L*_ (*p*_*i*_) refers to the highest score for all subcellular locations.

(13) $$S_{L}\left(p_{i}\right)=\max_{r\in C\left(p_{i}\right)}\left(OF\left(r\right)\right)$$

where *C*(*p*_*i*_) represents the subcellular location corresponding to the protein *p*_*i*_.

Finally, according to Eq. (11–13), the unique initial score *S*_*L*_(*p*_*i*_) of protein *p*_*i*_ is expressed as follows:

(14) $$S_{I}\left(p_{i}\right)=S_{H}\left(p_{i}\right)\times S_{L}\left(p_{i}\right)$$

### Computation of Ranking Scores

The ranking of protein *p*_*i*_ is called *S*_*F*_(*p*_*i*_), and $\sum_{p_{j}\in S_{CoN}\left(i\right)}P_{CoN}^{*}\left(p_{i},p_{j}\right)S_{F}\left(p_{j}\right)$ refers to the neighbor induction score. Based on this, the ranking score of each protein in the PPI network can be calculated by Eq. (15), as shown below:

(15) $$S_{F}\left(p_{i}\right)=\alpha \sum_{p_{j}\in S_{CoN}\left(i\right)}P_{CoN}^{*}\left(p_{i},p_{j}\right)S_{F}\left(p_{j}\right)+\left(1-\alpha \right)S_{I}\left(p_{i}\right)$$

Among them, the function of the parameter α (0 ≤ α < 1) is to adjust the weight of the two scores in the final ranking score. Based on the above analysis, the protein ranking score is a linear combination of its initial score and the neighborhood correlation score at the edge of the network. Therefore, formula (15) can be rewritten in matrix vector format as follows:

(16) $$S_{F}=\alpha *P_{CoN}^{*}*S_{F}+\left(1-\alpha \right)*S_{I}$$

In our study, the Jacobi iterative method is used to solve Eq. (16), as shown below:

(17) $$S_{F}^{t}=\alpha *P_{CoN}^{*}*S_{F}^{t-1}+\left(1-\alpha \right)*S_{I}$$

where $S_{F}^{t}$ is the protein’s scores obtained in the *t*th iteration.

Through the above analysis, we conclude that the overall framework of the NMTF-based model for the identification of essential protein (NTMEP) can be referred to as the following Algorithm 1.

**Algorithm 1. NTMEP**

**Input:** A PPI network *G*, subcellular localization information, homologous proteins information, stopping error ε, parameters *k*, α, and *K*

**Output:** Top *K* proteins sorted by *S*_*F*_ in descending order

Step 1: Calculate adjacency matrix **P*_*CoN*_* of the weighted PPI network according to Eq. (1)

Step 2: Reconstruct matrix **P*_*CoN*_* to $P_{CoN}^{*}$ by Eq. (2)–(10)

Step 3: Initialize initial vector *S*_*I*_ with *S*_*F*_^0^ = *S*_*I*_ and *t* = 0

Step 4: Compute *S_*F*_^*t*^* according Eq. (17)

Step 5. If | | *S*_*F*_^*t*^ – *S*_*F*_^*t*–1^| | < ε, then *PR S_*F*_* = *S_*F*_^*t*^* and terminate the algorithm. Otherwise, let *t* = *t*+1 and repeat Step 4

Step 6. Sort proteins by the value of *S*_*F*_ in the descending order

Step 7. Output top *K* of sorted proteins

## Results and Discussion

### Experimental Data

In the experiments, we use four data sets including protein–protein interaction set, experimentally verified essential protein set, subcellular location set, and homologous protein information set. We downloaded the relationships among proteins from the DIP database (Xenarios et al., 2002), which includes 1,167 essential proteins and a total of 24,743 interactions between 5,093 proteins after removing self-interactions and duplicate interactions. Also, these data are adopted to construct the weighted protein network based on the topological structures. The experimentally verified essential protein dataset with 1,285 essential proteins are derived from MIPS (Mewes et al., 2006), SGD (Cherry et al., 1998), DEG (Zhang and Lin, 2009), and SGDP (Saccharomyces Genome Deletion Project, 2012). From the COMPARTMENTS (Binder et al., 2014) database, we obtained the subcellular location data, which cover 11 categories (Endoplasmic, Nucleus, Cytoskeleton, Golgi, Cytosol, Vacuole, Plasma, Mitochondrion, Endosome, Peroxisome, and Extracellular) (Peng et al., 2015). The homologous protein information is collected come from the seventh edition of the InParanoid database (Ostlund et al., 2010) including paired comparisons of 100 whole genomes (99 eukaryotes and one prokaryote).

### Parameter α Sensitivity Analysis

In the NTMEP, the parameter α in Eq. (16), which used to weigh up the contribution of neighbor-induced score and initial score, was set to 0, 0.1, 0.2,…, and 1. While considering only the neighbor-induced score, α was set to 1. On the other hand, α was set to 0 when considering only the initial score. The impact of the parameter α to the performance of NTMEP is presented in Table 1. After the ranking scores of proteins were calculated with the different value of parameter α, we get the number of true essential proteins in the top 100, 200, 300, 400, 500, and 600 candidates, respectively. Table 1 shows that the performance of the NTMEP is very poor when α was set to 0 or 1. It can be seen from the data in Table 1 that the 0.1 and 0.2 groups have better prediction results. Especially, the best performance was achieved in the top 100 candidates when α was set to 0.1. Consequently, α was set to 0.2 in this article to make the NTMEP obtain good performance.

**TABLE 1:** The impact of parameter α to the performance of NTMEP.

	Top 100	Top 200	Top 300	Top 400	Top 500	Top 600
0	78	154	221	289	335	378
0.1	94	167	232	293	341	390
0.2	92	171	236	295	347	391
0.3	90	167	234	293	347	391
0.4	88	164	230	290	349	396
0.5	85	161	224	286	339	393
0.6	83	155	221	275	321	378
0.7	83	152	214	263	315	371
0.8	79	151	206	257	307	357
0.9	79	147	197	249	299	346
1	80	140	194	241	281	321

### Comprehensive Comparison With Other Methods

To comparatively study the performance of NTMEP in predicting essential proteins, we also implement 10 types of representative essential proteins prediction methods, like DC (Joy et al., 2014), IC (Estrada and Rodríguez-Velázquez, 2005), CC (Wang et al., 2012), BC (Li et al., 2018), SC (Tew et al., 2007), NC (Zhang et al., 2018), PeC (Li et al., 2012), CoEWC (Zhang et al., 2013), POEM (Zhao et al., 2014), and JDC (Zhong et al., 2021), which are state-of-the-art prediction methods for the well essential protein prediction.

**FIGURE 1:**
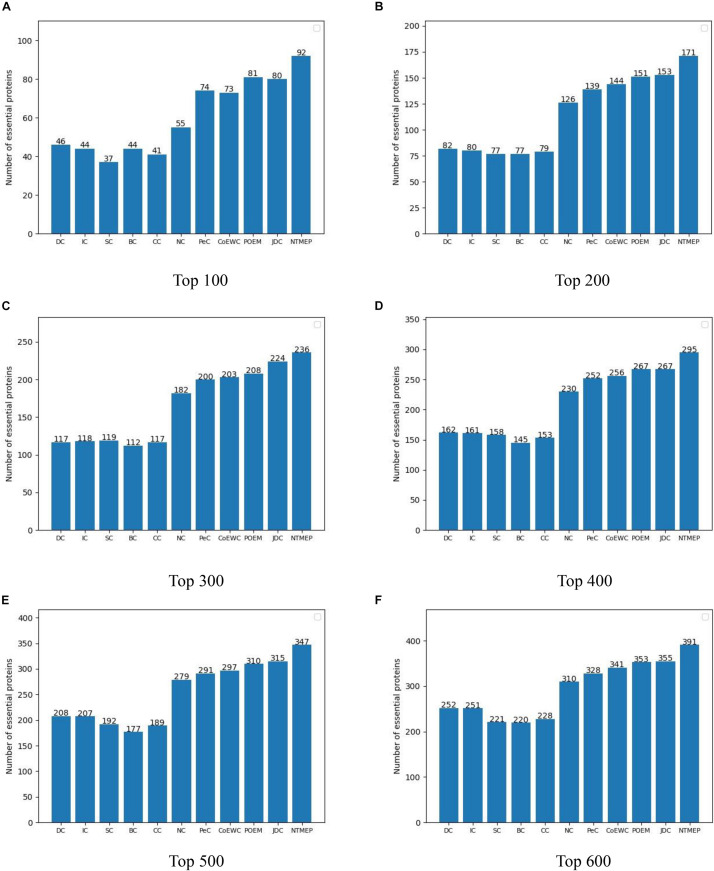
Number of actual essential proteins identified by NTMEP and other ten previously competitive methods at various *k* values. **(A)** Top 100 ranked proteins; **(B)** Top 200 ranked proteins; **(C)** Top 300 ranked proteins; **(D)** Top 400 ranked proteins; **(E)** Top 500 ranked proteins; **(F)** Top 600 ranked proteins.

The higher number of essential proteins within the top *k* of the ranking list means the more real essential proteins are predicted successfully. Parameter *k*, which is set to 100, 200, 300, 400, 500, and 600, denotes the number of essential protein candidates selected. The number of real essential proteins within top *k* candidates is shown in Figure 1. NTMEP consistently outperformed the other competitive methods at various *k* cutoffs and ranked 92, 85.5, 78.7, 73.8, 69.4, and 65.2% of positive samples in top 100, 200, 300, 400, 500, and 600, respectively. Especially, as for the top 100 of essential protein candidates, NTMEP has higher predict accuracy 46, 48, 55, 48, 51, 37, 18, 19, 11, and 12% than that obtained from DC, IC, CC, BC, SC, NC, PeC, CoEWC, POEM, and JDC, respectively. In those competitive methods, JDC had the best accuracy and ranked 80, 76.5, 74.7, 66.8, 63, and 59.2% in the top 100–600, respectively. Compared with JDC, NTMEP improved by 15% in top 100, 11.8% in top 200, 5.4% in top 300, 10.5% in top 400, 10.2% in top 500, and 10.1% in top 600.

### Validated by Precision–Recall Curves

**FIGURE 2:**
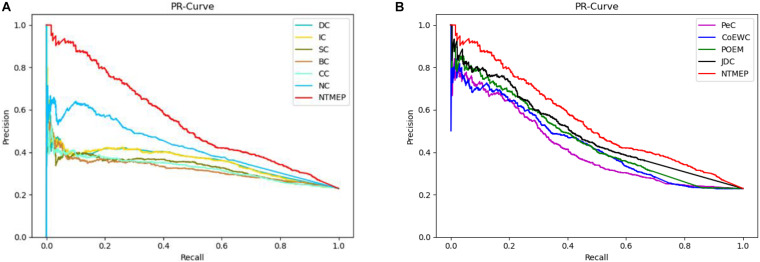
Performance comparison between NTMEP and other ten methods in terms of PR curves. **(A)** PR curves of DC, IC, SC, BC, CC, NC, and NTMEP; **(B)** PR curves of PeC, CoEWC, POEM, JDC, and NTMEP.

To obtain a fair and convincing comparison, the precision–recall (PR) curve is used to evaluate the prediction performance for essential proteins of our method and other state-of-the-art methods. The value of cutoffs, presented as *k*, is ranged from 1 to 5,093. We compute the scores of all proteins by using each algorithm and sorted it in descending order, respectively. The top *k* proteins are selected as a positive set, namely, essential protein candidates, and others as the negative set, namely, non-essential protein candidates. Figure 2 compares the results obtained from the different methods. As shown in Figure 2A, compared with DC, IC, BC, CC, SC, and NC, the PR curves of NTMEP reported significantly higher capability for identifying essential proteins. The results obtained from our method and PeC, CoEWC, POEM, and JDC are presented in Figure 2B. Looking at Figure 2B, in the first part of the PR curve, it is apparent that the precision of our method has the best performance compared to those methods. In order to give quantitative comparison results, the area under the curve (AUC) values of the PR curve were computed, respectively, as shown in Table 2. As a whole, the NTMEP dramatically outperformed those competitive methods.

**TABLE 2:** The AUC values of the PR curve obtained from NTMEP and other 10 competitive methods.

Method	NTMEP	DC	IC	SC	BC	CC
**AUC value of PR curve**	0.549	0.359	0.357	0.331	0.319	0.326
		
		**NC**	**PeC**	**CoEWC**	**POEM**	**JDC**
		
		0.425	0.492	0.463	0.439	0.417

### Validated by Jackknife Methodology

**FIGURE 3:**
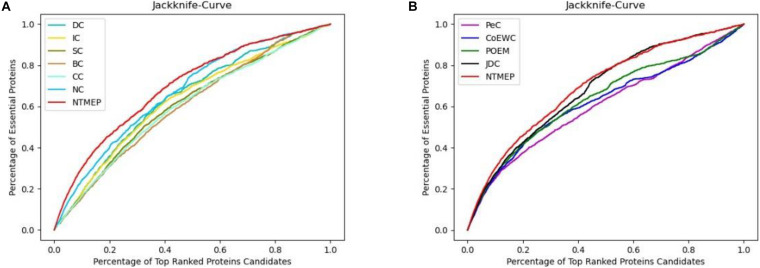
Performance comparison between NTMEP and other ten methods in terms of Jackknife curves. **(A)** Jackknife curves of DC, IC, SC, BC, CC, NC, and NTMEP; **(B)** Jackknife curves of PeC, CoEWC, POEM, JDC, and NTMEP.

In this subsection, we employ the jackknife curves to assess the performance of our NTMEP method and other existing methods (DC, BC, CC, SC, IC, NC, PeC, CoEWC, POEM, and JDC), the various top number of ranked proteins as candidates. The jackknife curves of all the methods are displayed in Figure 3, where the horizontal axis denotes the number of proteins ranked at the top in descending order with each corresponding method, and the vertical axis is the accumulative quantity of the real essential proteins within the ranked proteins. Figures 3A,B illustrate the jackknife curves of all the competitive methods compared with NTMEP, respectively. As is seen from Figure 3A, the curve of NTMEP reported a higher number of real essential proteins than other existing centrality measure methods, such as DC, BC, CC, SC, IC, and NC. As shown in Figure 3B, NTMEP is also better than PeC, CoEWC, POEM, and JDC. To give quantitative comparison results, the AUC values of jackknife curve were computed, respectively, as shown in Table 3. From Figure 3 and Table 3, it is clear that the NTMEP method outperforms the other 10 essential protein prediction methods.

**TABLE 3:** The AUC values of jackknife curve obtained from NTMEP and other 10 methods.

Method	NTMEP	DC	IC	SC	BC	CC
**AUC value of jackknife curve**	0.697	0.640	0.628	0.607	0.601	0.600
		
		**NC**	**PeC**	**CoEWC**	**POEM**	**JDC**
		
		0.670	0.603	0.618	0.635	0.684

In summary, these results demonstrated the powerful ability of NTMEP in identifying essential proteins. This finding is reasonable because our method adopts NMTF to find the potential interactions between proteins, which could provide additional interaction information and help to improve the prediction results by a large margin.

### Analysis of the Differences Between NTMEP and Other 10 Competitive Prediction Methods

This subsection will analyze the difference between NTMEP and other prediction methods through experimental results. Firstly, 11 protein sets were constructed by NTMEP and other 10 prediction methods (DC, IC, CC, BC, SC, NC, PeC, CoEWC, POEM, and JDC), and each protein set contains the top 100 essential proteins predicted by each prediction method. The number of proteins that overlap between the NTMEP method and other methods and the number of proteins that differ are shown in Table 4.

**TABLE 4:** Comparison of the overlap and difference of the top 100 proteins identified by NTMEP and other 10 methods.

Methods (Mi)	| Mi∩NTMEP|	| NTMEP-Mi| and | Mi-NTMEP|	Number of essential proteins in {Mi-NTMEP}	Number of essential proteins in {NTMEP-Mi }	Percentage of essential proteins in {Mi-NTMEP}	Percentage of essential proteins in {NTMEP-Mi}
DC	15	85	32	78	37.6%	91.8%
IC	15	85	30	78	35.3%	91.8%
SC	12	88	25	80	28.4%	90.9%
BC	11	89	34	82	38.2%	92.1%
CC	13	87	29	80	33.3%	92.0%
NC	33	67	25	62	37.3%	92.5%
PeC	44	56	33	50	58.9%	91.1%
CoEWC	46	54	30	49	55.6%	90.7%
POEM	49	51	35	46	68.6%	90.2%
JDC	40	60	42	54	70.0%	90.0%

In Table 4, Mi refers to one of the 10 prediction methods (DC, IC, CC, BC, SC, NC, PeC, CoEWC, POEM, and JDC); |Mi∩NTMEP| represents the number of common proteins predicted by both Mi and NTMEP in the top 100 ranked proteins. {Mi-NTMEP} refers to the difference set in the top 100 ranked proteins, while proteins were selected as essential proteins by Mi but not by NTMEP. Moreover, | Mi-NTMEP| represents the number of proteins in the difference set. Similarly, {NTMEP-Mi} denotes the difference set constituted by the proteins belonging to NTMEP but not to Mi, and the number is denoted by |NTMEP-Mi|.

As shown in Table 4, the second row of the table shows that 85 essential protein candidates out of the top 100 essential protein candidates predicted by DC are different from those predicted by NTMEP, while 32 of these 85 predicted essential protein candidates are true essential proteins; thus, the percentage of essential proteins in the difference set is 37.6%. Among the top 100 essential protein candidates predicted by NTMEP, 85 essential protein candidates were different from those predicted by DC, but 78 of them were accurate; thus, the percentage of essential proteins in the difference set was 91.8%. From this line of data, it can be seen that most of the top 100 essential protein candidates predicted by NTMEP are different from those candidates predicted by DC. Moreover, NTMEP predicts far more true key proteins than DC. This indicates that NTMEP not only is a different method from DC but also shows that NTMEP is much better than DC in distinguishing essential proteins from common proteins. Similarly, it can be seen from the other rows of the table that NTMEP maintains this advantage over all other prediction methods.

## Conclusion

In reviewing the literature, previous studies developed many computational methods to predict essential proteins effectively. However, these methods do not take full account of the false-positive and -negative noise generated from high-throughput experimentation and the process of the weighted PPI network construction. To get the utmost out of the complex association between proteins, NMTF is introduced into our proposed method. Moreover, subcellular localization and homologous protein information are used in the final scoring stage instead of the stage of establishing the weighted network. Also, a comprehensive experiment is carried out and the results show that our new method can obtain a better performance compared with other methods. A possible explanation for these results might be that there are deep relationships between proteins which are not founded by high-throughput experimentation, and fusion of multiple data raises the cost and reduces the overall efficiency of the process. These results add to the rapidly expanding field of computational methods for predicting essential proteins. It is unfortunate that the study did not solve the problem of noise generated by multisource data fusion. This is an important issue for future research.

## Data Availability Statement

The datasets presented in this study can be found in online repositories. The names of the repository/repositories and accession number(s) can be found in the article/supplementary material.

## Author Contributions

ZZ and MJ obtained the protein–protein interaction data, benchmark essential protein dataset, subcellular location data, and homologous protein information. ZZ, MJ, and XQ designed the new method, NTMEP, and analyzed the results. ZZ, DW, and WZ drafted and revised the manuscript together. All authors have read and approved the manuscript.

## Conflict of Interest

The authors declare that the research was conducted in the absence of any commercial or financial relationships that could be construed as a potential conflict of interest.

## Publisher’s Note

All claims expressed in this article are solely those of the authors and do not necessarily represent those of their affiliated organizations, or those of the publisher, the editors and the reviewers. Any product that may be evaluated in this article, or claim that may be made by its manufacturer, is not guaranteed or endorsed by the publisher.

## References

[B1] Binder J. X. Pletscher-Frankild S. Tsafou K. Stolte C. O’Donoghue S. I. Schneider R. (2014). COMPARTMENTS: unification and visualization of protein subcellular localization evidence. *Database (Oxford)* 2014:bau012. 10.1093/database/bau012 24573882 PMC3935310

[B2] Björnsdottir K. (2001). Language, research and nursing practice. *J. Adv. Nurs.* 33 159–166. 10.1111/j.1365-2648.2001.01648.x 11168697

[B3] Cherry J. M. Adler C. Ball C. Chervitz S. A. Dwight S. S. Hester E. T. (1998). SGD: *Saccharomyces* genome database. *Nucleic Acids Res.* 26 73–79. 10.1093/nar/26.1.73 9399804 PMC147204

[B4] Estrada E. Rodríguez-Velázquez J. A. (2005). Subgraph centrality in complex networks. *Phys. Rev. E Stat. Nonlin. Soft. Matter. Phys.* 71:056103. 10.1103/PhysRevE.71.056103 16089598

[B5] Glass J. I. Hutchison C. A. Smith H. O. Venter J. C. (2009). A systems biology tour de force for a near-minimal bacterium. *Mol. Syst. Biol*. 5:330. 10.1038/msb.2009.89 19953084 PMC2824490

[B6] Hahn M. W. Kern A. D. (2005). Comparative genomics of centrality and essentiality in three eukaryotic protein-interaction networks. *Mol. Biol. Evol.* 22 803–806. 10.1093/molbev/msi072 15616139

[B7] Hernando A. Bobadilla J. Ortega F. (2016). A non negative matrix factorization for collaborative filtering recommender systems based on a Bayesian probabilistic model. *Knowl. Based Syst.* 97 188–202. 10.1016/j.knosys.2015.12.018

[B8] Hua W. Nie F. Huang H. Makedon F. (2011). “Fast nonnegative matrix Tri-factorization for large-scale data co-clustering,” in *Proceedings of the 22nd International Joint Conference on Artificial Intelligence. DBLP*, 2011, (Barcelona, CA).

[B9] Joy M. P. Brock A. Ingber D. E. Huang S. (2014). High-betweenness proteins in the yeast protein interaction network. *J. Biomed. Biotechnol.* 2005 96–103. 10.1155/JBB.2005.96 16046814 PMC1184047

[B10] Kobayashi K. Ehrlich S. D. Albertini A. Amati G. Andersen K. K. Arnaud M. (2003). Essential *Bacillus subtilis* genes. *Proc. Natl. Acad. Sci. U.S.A.* 100 4678–4683. 10.1073/pnas.0730515100 12682299 PMC153615

[B11] Lee D. D. Seung H. S. (1999). Learning the parts of objects by non-negative matrix factorization. *Nature* 401 788–791. 10.1038/44565 10548103

[B12] Lei X. Fang M. Wu F. X. Chen L. (2018). Improved flower pollination algorithm for identifying essential proteins. *BMC Syst. Biol.* 12:46. 10.1186/s12918-018-0573-y 29745838 PMC5998882

[B13] Lei X. Yang X. Fujita H. (2019). Random walk based method to identify essential proteins by integrating network topology and biological characteristics. *Knowl. Based Syst.* 167 53–67. 10.1016/j.knosys.2019.01.012

[B14] Li G. Li M. Wang J. X. Li Y. Pan Y. (2018). United neighborhood closeness centrality and orthology for predicting essential proteins. *IEEE ACM Trans. Comput. Biol. Bioinform.* 17 1451–1458. 10.1109/TCBB.2018.2889978 30596582

[B15] Li G. Li M. Wang J. X. Wu J. Wu F. X. Pan Y. (2016). Predicting essential proteins based on subcellular localization, orthology and PPI networks. *BMC Bioinformatics* 17:279. 10.1186/s12859-016-1115-5 27586883 PMC5009824

[B16] Li M. Zhang H. Wang J. X. Pan Y. (2012). A new essential protein discovery method based on the integration of protein-protein interaction and gene expression data. *BMC Syst. Biol.* 6:15. 10.1186/1752-0509-6-15 22405054 PMC3325894

[B17] Luo X. Zhou M. Li S. You Z. Xia Y. Zhu Q. (2016). A nonnegative latent factor model for large-scale sparse matrices in recommender systems via alternating direction method. *IEEE Trans. Neural Netw. Learn. Syst.* 27 579–592. 10.1109/TNNLS.2015.2415257 26011893

[B18] Mewes H. W. Frishman D. Mayer K. F. X. Munsterkotter M. Noubibou O. Pagel P. (2006). MIPS: analysis and annotation of proteins from whole genomes in 2005. *Nucleic Acids Res.* 34 D169–D172. 10.1093/nar/gkj148 16381839 PMC1347510

[B19] Ostlund G. Schmitt T. Forslund K. Köstler T. Messina D. N. Roopra S. (2010). InParanoid 7: new algorithms and tools for eukaryotic orthology analysis. *Nucleic Acids Res.* 38 D196–D203. 10.1093/nar/gkp931 19892828 PMC2808972

[B20] Peng W. Wang J. X. Wang W. Liu Q. Wu F. X. Pan Y. (2012). Iteration method for predicting essential proteins based on orthology and protein-protein interaction networks. *BMC Syst. Biol.* 6:87. 10.1186/1752-0509-6-87 22808943 PMC3472210

[B21] Peng X. Wang J. Zhong J. Luo J. Pan Y. (2015). “An efficient method to identify essential proteins for different species by integrating protein subcellular localization information,” in *Proceedings of the 2015 IEEE International Conference on Bioinformatics and Biomedicine (BIBM)*, (Washington, DC), 277–280.

[B22] Ren J. Wang J. X. Li M. Wang H. Liu B. (2011). Prediction of essential proteins by integration of PPI network topology and protein complexes information. *Bioinform. Res. Appl.* 6674 12–24. 10.1007/978-3-642-21260-4_6

[B23] Saccharomyces Genome Deletion Project (2012). *Saccharomyces Genome Deletion Project.* Available online at: http://yeastdeletion.stanford.edu/ (accessed June 20, 2012).

[B24] Tang X. Li X. Hu S. Zhao B. (2018). A framework for identifying functional modules in dynamic networks. *Int. J. Data Mining Bioinform.* 21 1–17. 10.1504/IJDMB.2018.095554

[B25] Tew K. L. Li X. L. Tan S. H. (2007). Functional centrality: detecting lethality of proteins in protein interaction networks. *Genome Inform.* 19 166–177. 18546514

[B26] Wang J. X. Li M. Wang H. Pan Y. (2012). Identification of essential proteins based on edge clustering coefficient. *IEEE ACM Trans. Comput. Biol. Bioinform.* 9 1070–1080. 10.1109/TCBB.2011.147 22084147

[B27] Wuchty S. Stadler P. F. (2003). Centers of complex networks. *J. Theor. Biol.* 223 45–53. 10.1016/s0022-5193(03)00071-7 12782116

[B28] Xenarios I. Salwínski L. Duan X. J. Higney P. Kim S. M. Eisenberg D. (2002). DIP, the database of interacting proteins: a research tool for studying cellular networks of protein interactions. *Nucleic Acids Res.* 30 303–305. 10.1093/nar/30.1.303 11752321 PMC99070

[B29] Xi J. Li A. Wang M. (2018). A novel unsupervised learning model for detecting driver genes from pan-cancer data through matrix tri-factorization framework with pairwise similarities constraints. *Neurocomputing* 296 64–73. 10.1016/j.neucom.2018.03.026

[B30] Zhang F. Peng W. Yang Y. Dai W. Song J. (2019). A novel method for identifying essential genes by fusing dynamic protein-protein interactive networks. *Genes (Basel)* 10:31. 10.3390/genes10010031 30626157 PMC6356314

[B31] Zhang R. Lin Y. (2009). DEG 5.0, a database of essential genes in both prokaryotes and eukaryotes. *Nucleic Acids Res.* 37 D455–D458. 10.1093/nar/gkn858 18974178 PMC2686491

[B32] Zhang W. Xu J. Li Y. Zou X. (2018). Detecting essential proteins based on network topology, gene expression data, and gene ontology information. *IEEE ACM Trans. Comput. Biol. Bioinform.* 15 109–116. 10.1109/TCBB.2016.2615931 28650821

[B33] Zhang X. Xu J. Xiao W. X. (2013). A new method for the discovery of essential proteins. *PLoS One* 8:e58763. 10.1371/journal.pone.0058763 23555595 PMC3605424

[B34] Zhao B. H. Wang J. X. Li M. Wu F. X. Pan Y. (2014). Prediction of essential proteins based on overlapping essential modules. *IEEE Trans. Nanobiosci.* 13 415–424. 10.1109/TNB.2014.2337912 25122840

[B35] Zhao B. H. Wang J. X. Li X. Wu F. X. (2016). Essential protein discovery based on a combination of modularity and conservatism. *Methods* 110 54–63. 10.1016/j.ymeth.2016.07.005 27402354

[B36] Zhong J. Tang C. Peng W. Xie M. Sun Y. Tang Q. (2021). A novel essential protein identification method based on PPI networks and gene expression data. *BMC Bioinformatics* 22:248. 10.21203/rs.3.rs-55902/v2 PMC8120700 33985429

[B37] Žitnik M. Janjić V. Larminie C. Zupan B. Pržulj N. (2013). Discovering disease-disease associations by fusing systems-level molecular data. *Sci. Rep.* 3:3202. 10.1038/srep03202 24232732 PMC3828568

